# 2a,3a-Dihydroxy­androstan-16-one

**DOI:** 10.1107/S1600536809029493

**Published:** 2009-07-31

**Authors:** Li Zhang, Xin Fang, Xiao-Jiang Hao, Yang Lu

**Affiliations:** a Institute of Materia Medica, Chinese Academy of Medical Sciences, and Peking Union Medical College, 1 Xiannong Tan Street, Beijing 100050, People’s Republic of China; b State Key Laboratory of Phytochemistry and Plant Resources in West China, Kunming Institute of Botany, Chinese Academy of Sciences, Kunming 650204, Yunnan, People’s Republic of China

## Abstract

The title compound, C_19_H_28_O_4_, is a new androstane steroid derivative. In the crystal, mol­ecules are linked along the *a* axis by inter­molecular O—H⋯O hydrogen bonds.

## Related literature

The title compound was obtained from the methanol extract of stems of *Trichilia claussenii* by column chromatograph, see: Pupo *et al.* (1997 ▶). It shows strong insecticidal activity, see: Champagne *et al.* (1992 ▶). 
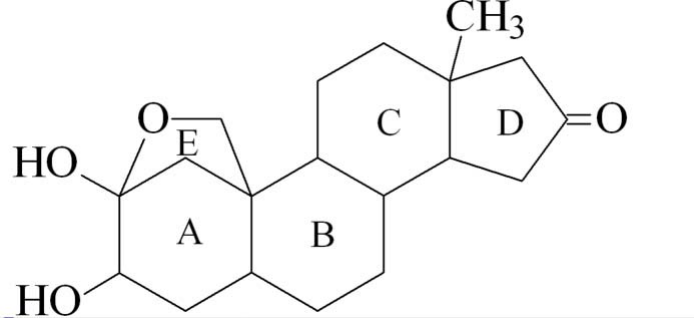


## Experimental

### 

#### Crystal data

C_19_H_28_O_4_
*M* *_r_* = 320.41
Monoclinic, 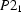

*a* = 10.8687 (2) Å
*b* = 6.3379 (1) Å
*c* = 12.9038 (2) Å
β = 112.882 (1)°
*V* = 818.93 (2) Å^3^
*Z* = 2
Mo *K*α radiation
μ = 0.09 mm^−1^
*T* = 295 K
0.20 × 0.20 × 0.20 mm

#### Data collection

MAC DIP 2030K diffractometer
Absorption correction: none
2065 measured reflections
2065 independent reflections
1929 reflections with *I* > 2σ(*I*)
*R*_int_ = 0.015

#### Refinement

*R*[*F* ^2^ > 2σ(*F* ^2^)] = 0.038
*wR*(*F* ^2^) = 0.105
*S* = 1.14
2065 reflections
208 parameters
1 restraint
H-atom parameters constrained
Δρ_max_ = 0.26 e Å^−3^
Δρ_min_ = −0.15 e Å^−3^

### 

Data collection: *DENZO* (Otwinowski & Minor, 1997 ▶); cell refinement: *SCALEPACK* (Otwinowski & Minor, 1997 ▶); data reduction: *SCALEPACK*; program(s) used to solve structure: *SHELXS97* (Sheldrick, 2008 ▶); program(s) used to refine structure: *SHELXL97* (Sheldrick, 2008 ▶); molecular graphics: *PLATON* (Spek, 2009 ▶); software used to prepare material for publication: *SHELXL97*.

## Supplementary Material

Crystal structure: contains datablocks I, global. DOI: 10.1107/S1600536809029493/jh2087sup1.cif

Structure factors: contains datablocks I. DOI: 10.1107/S1600536809029493/jh2087Isup2.hkl

Additional supplementary materials:  crystallographic information; 3D view; checkCIF report

## Figures and Tables

**Table 1:** Hydrogen-bond geometry (Å, °)

*D*—H⋯*A*	*D*—H	H⋯*A*	*D*⋯*A*	*D*—H⋯*A*
O2—H2*A*⋯O1^i^	0.82	2.03	2.8088 (19)	158

Symmetry code: (i) 
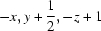
.

**Fig. 1.**
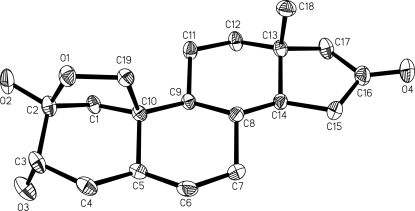
View of the molecule showing the atom-labelling scheme. Displacement ellipsoids are drawn at the 30% probability level.

**Fig. 2.**
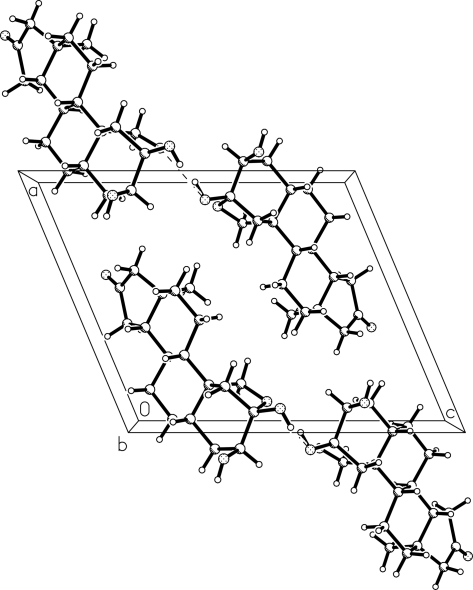
The molecular packing of (I) viewed down the b axis.

## supplementary crystallographic information

### Comment

The title compound,(I), was yielded from the methanol extract of stems of Trichilia claussenii by column chromatographies (Pupo *et al.*, 1997) and recrystasllized from methanol-hexane(1:1). As it shows strong biological activities against insects (Champagne *et al.*, 1992) we have determined its crystal stucture, Fig. 1, Table 1.

In this structure, rings A,B and C adopt chair conformations, and both ring D,*E* adopt envelope conformations. The dihedral angle between the least-squares plane through the 6 atoms of rings A and B is 9.2 (1)°, and that between rings B and C and that between rings C and D are 175.0 (2)° and 2.5 (1)°, respectively.

Besides, the molecules of the title compound are linked into each other along *a* axis, by intermolecular hydrogen bonds O—H···O. Atom O2 acts as hydrogen-bond donor to atom O1 at (-*x*, *y* + 1/2, -*z* + 1). Molecules pack in ribbons along the *b* axis, Fig. 2.

### Experimental

The title compound was prepared according to the procedure of extracting Trichilia claussenii (Pupo *et al.*, 1997). At the temprature of 283 K and unventilated condition, single crystals of (I) were obtained from mixed solvent of methanol-hexane(1:1) within two weeks.

### Refinement

In the absence of significant anomalous dispersion effects, Freidel pairs were merged. The position of the hydroxy H atoms were refined freely along with an isotropic displacement parameter *U*_iso_(H)= 1.2*U*_eq_(C). The methyl H atoms were then constrained to an ideal geometry with C—H distances of 0.96 Å and *U*_iso_(H) = 1.5*U*_eq_(C), but each group was allowed to rotate freely about its C—C bond. All the other H atoms were placed in geometrically idealized positions and constrained to ride on their parent atoms with C—H distances in the range of 0.92–0.98 Å and *U*_iso_(H) = 1.2*U*_eq_(C).

### Crystal data

**Table:**

C_19_H_28_O_4_	*F*(000) = 348
*M**_r_* = 320.41	*D*_x_ = 1.299 Mg m^−^^3^
Monoclinic, *P*2_1_	Mo *K*α radiation, λ = 0.71073 Å
Hall symbol: P 2yb	Cell parameters from 2065 reflections
*a* = 10.8687 (2) Å	θ = 1.7–27.6°
*b* = 6.3379 (1) Å	µ = 0.09 mm^−^^1^
*c* = 12.9038 (2) Å	*T* = 295 K
β = 112.882 (1)°	Block, colourless
*V* = 818.93 (2) Å^3^	0.20 × 0.20 × 0.20 mm
*Z* = 2	

### Data collection

**Table:**

MAC DIP 2030K diffractometer	1929 reflections with *I* > 2σ(*I*)
Radiation source: rotate anode	*R*_int_ = 0.015
graphite	θ_max_ = 27.6°, θ_min_ = 1.7°
Detector resolution: 0 pixels mm^-1^	*h* = −14→13
ω scans	*k* = 0→8
2065 measured reflections	*l* = 0→16
2065 independent reflections	

### Refinement

**Table:**

Refinement on *F*^2^	Primary atom site location: structure-invariant direct methods
Least-squares matrix: full	Secondary atom site location: difference Fourier map
*R*[*F*^2^ > 2σ(*F*^2^)] = 0.038	Hydrogen site location: inferred from neighbouring sites
*wR*(*F*^2^) = 0.105	H-atom parameters constrained
*S* = 1.14	*w* = 1/[σ^2^(*F*_o_^2^) + (0.0593*P*)^2^ + 0.0933*P*] where *P* = (*F*_o_^2^ + 2*F*_c_^2^)/3
2065 reflections	(Δ/σ)_max_ < 0.001
208 parameters	Δρ_max_ = 0.26 e Å^−^^3^
1 restraint	Δρ_min_ = −0.15 e Å^−^^3^

### Special details

**Table:**

Geometry. All e.s.d.'s (except the e.s.d. in the dihedral angle between two l.s. planes) are estimated using the full covariance matrix. The cell e.s.d.'s are taken into account individually in the estimation of e.s.d.'s in distances, angles and torsion angles; correlations between e.s.d.'s in cell parameters are only used when they are defined by crystal symmetry. An approximate (isotropic) treatment of cell e.s.d.'s is used for estimating e.s.d.'s involving l.s. planes.
Refinement. Refinement of *F*^2^ against ALL reflections. The weighted *R*-factor *wR* and goodness of fit *S* are based on *F*^2^, conventional *R*-factors *R* are based on *F*, with *F* set to zero for negative *F*^2^. The threshold expression of *F*^2^ > σ(*F*^2^) is used only for calculating *R*-factors(gt) *etc*. and is not relevant to the choice of reflections for refinement. *R*-factors based on *F*^2^ are statistically about twice as large as those based on *F*, and *R*- factors based on ALL data will be even larger.

### Fractional atomic coordinates and isotropic or equivalent isotropic displacement parameters (Å^2^)

**Table:**

	*x*	*y*	*z*	*U*_iso_*/*U*_eq_	
O1	0.10604 (14)	0.6665 (3)	0.44798 (11)	0.0387 (3)	
O2	0.09261 (16)	1.0230 (3)	0.48050 (15)	0.0530 (5)	
H2A	0.0221	1.0426	0.4882	0.080*	
O3	−0.09685 (18)	1.0625 (3)	0.25157 (18)	0.0597 (5)	
H3A	−0.1755	1.0667	0.2083	0.089*	
O4	0.61221 (19)	0.0525 (4)	0.09632 (16)	0.0657 (5)	
C1	0.17314 (18)	0.9035 (3)	0.34250 (16)	0.0321 (4)	
H1A	0.2610	0.9394	0.3976	0.038*	
H1B	0.1443	1.0113	0.2846	0.038*	
C2	0.07349 (19)	0.8750 (3)	0.39679 (18)	0.0371 (4)	
C3	−0.0693 (2)	0.8638 (4)	0.3084 (2)	0.0423 (5)	
H3B	−0.1314	0.8411	0.3457	0.051*	
C4	−0.08066 (18)	0.6822 (4)	0.22815 (18)	0.0419 (5)	
H4B	−0.1622	0.6999	0.1618	0.050*	
H4C	−0.0886	0.5516	0.2642	0.050*	
C5	0.03684 (17)	0.6613 (4)	0.19065 (15)	0.0337 (4)	
H5A	0.0300	0.7763	0.1379	0.040*	
C6	0.02645 (19)	0.4533 (4)	0.12841 (17)	0.0418 (5)	
H6A	0.0208	0.3383	0.1760	0.050*	
H6B	−0.0550	0.4533	0.0611	0.050*	
C7	0.14492 (19)	0.4159 (4)	0.09552 (16)	0.0425 (5)	
H7A	0.1436	0.5195	0.0398	0.051*	
H7B	0.1371	0.2771	0.0618	0.051*	
C8	0.27756 (17)	0.4311 (3)	0.19700 (14)	0.0294 (4)	
H8A	0.2807	0.3202	0.2509	0.035*	
C9	0.28860 (17)	0.6477 (3)	0.25428 (14)	0.0278 (4)	
H9	0.2777	0.7530	0.1958	0.033*	
C10	0.17217 (16)	0.6848 (3)	0.29237 (14)	0.0273 (4)	
C11	0.42783 (17)	0.6878 (4)	0.34704 (16)	0.0357 (4)	
H11A	0.4325	0.8331	0.3720	0.043*	
H11B	0.4390	0.5978	0.4109	0.043*	
C12	0.54289 (19)	0.6468 (3)	0.30905 (19)	0.0383 (5)	
H12A	0.5399	0.7492	0.2523	0.046*	
H12B	0.6275	0.6625	0.3725	0.046*	
C13	0.53218 (18)	0.4254 (3)	0.26115 (15)	0.0314 (4)	
C14	0.39575 (18)	0.4035 (3)	0.16258 (15)	0.0311 (4)	
H14	0.3903	0.5191	0.1105	0.037*	
C15	0.4097 (2)	0.2001 (4)	0.10396 (17)	0.0429 (5)	
H15A	0.3818	0.0783	0.1349	0.051*	
H15B	0.3572	0.2069	0.0237	0.051*	
C16	0.5576 (2)	0.1902 (5)	0.12818 (18)	0.0455 (5)	
C17	0.6267 (2)	0.3747 (4)	0.2021 (2)	0.0451 (5)	
H17A	0.6348	0.4934	0.1577	0.054*	
H17B	0.7147	0.3357	0.2558	0.054*	
C18	0.5558 (2)	0.2595 (4)	0.35272 (18)	0.0428 (5)	
H18A	0.4971	0.2857	0.3910	0.064*	
H18B	0.6468	0.2667	0.4056	0.064*	
H18C	0.5382	0.1217	0.3192	0.064*	
C19	0.17183 (18)	0.5448 (3)	0.39026 (15)	0.0314 (4)	
H19A	0.1239	0.4144	0.3617	0.038*	
H19B	0.2624	0.5108	0.4406	0.038*	

### Atomic displacement parameters (Å^2^)

**Table:**

	*U*^11^	*U*^22^	*U*^33^	*U*^12^	*U*^13^	*U*^23^
O1	0.0390 (7)	0.0436 (8)	0.0434 (7)	0.0018 (7)	0.0270 (6)	0.0048 (7)
O2	0.0462 (9)	0.0557 (11)	0.0714 (10)	−0.0022 (8)	0.0384 (8)	−0.0216 (9)
O3	0.0445 (9)	0.0469 (10)	0.0889 (13)	0.0149 (8)	0.0274 (9)	0.0156 (10)
O4	0.0664 (11)	0.0689 (13)	0.0684 (11)	0.0217 (10)	0.0334 (9)	−0.0171 (10)
C1	0.0285 (8)	0.0285 (10)	0.0453 (10)	−0.0002 (7)	0.0209 (8)	0.0004 (8)
C2	0.0349 (10)	0.0361 (11)	0.0491 (11)	0.0015 (8)	0.0260 (9)	−0.0001 (9)
C3	0.0313 (10)	0.0423 (12)	0.0615 (13)	0.0066 (9)	0.0271 (10)	0.0073 (11)
C4	0.0241 (9)	0.0481 (12)	0.0516 (11)	−0.0003 (9)	0.0128 (8)	0.0063 (11)
C5	0.0270 (8)	0.0364 (10)	0.0349 (8)	0.0024 (8)	0.0088 (7)	0.0054 (9)
C6	0.0275 (9)	0.0485 (13)	0.0389 (10)	−0.0027 (9)	0.0015 (8)	−0.0040 (9)
C7	0.0374 (10)	0.0499 (13)	0.0332 (9)	0.0025 (10)	0.0062 (8)	−0.0093 (10)
C8	0.0299 (8)	0.0310 (9)	0.0274 (8)	−0.0005 (7)	0.0113 (7)	−0.0021 (7)
C9	0.0274 (8)	0.0281 (9)	0.0321 (8)	0.0000 (7)	0.0160 (7)	0.0019 (7)
C10	0.0249 (8)	0.0268 (8)	0.0326 (8)	0.0000 (7)	0.0138 (6)	0.0033 (7)
C11	0.0277 (8)	0.0367 (10)	0.0460 (10)	−0.0052 (8)	0.0177 (8)	−0.0122 (9)
C12	0.0307 (9)	0.0337 (11)	0.0563 (12)	−0.0050 (8)	0.0235 (9)	−0.0087 (9)
C13	0.0309 (9)	0.0295 (9)	0.0386 (9)	−0.0002 (8)	0.0187 (8)	−0.0001 (8)
C14	0.0377 (9)	0.0296 (10)	0.0289 (8)	0.0043 (8)	0.0162 (7)	0.0018 (8)
C15	0.0490 (11)	0.0444 (12)	0.0359 (9)	0.0057 (11)	0.0173 (9)	−0.0081 (9)
C16	0.0533 (12)	0.0482 (13)	0.0422 (10)	0.0127 (11)	0.0264 (9)	0.0016 (11)
C17	0.0426 (11)	0.0412 (12)	0.0630 (13)	0.0060 (10)	0.0331 (10)	−0.0001 (11)
C18	0.0453 (12)	0.0409 (11)	0.0363 (10)	0.0043 (9)	0.0093 (9)	0.0064 (9)
C19	0.0308 (8)	0.0320 (10)	0.0352 (9)	0.0006 (8)	0.0171 (7)	0.0054 (8)

### Geometric parameters (Å, °)

**Table:**

O1—C19	1.440 (2)	C8—H8A	0.9800
O1—C2	1.458 (3)	C9—C11	1.542 (2)
O2—C2	1.384 (3)	C9—C10	1.543 (2)
O2—H2A	0.8200	C9—H9	0.9800
O3—C3	1.429 (3)	C10—C19	1.545 (2)
O3—H3A	0.8200	C11—C12	1.533 (2)
O4—C16	1.214 (3)	C11—H11A	0.9700
C1—C2	1.512 (2)	C11—H11B	0.9700
C1—C10	1.528 (3)	C12—C13	1.519 (3)
C1—H1A	0.9700	C12—H12A	0.9700
C1—H1B	0.9700	C12—H12B	0.9700
C2—C3	1.529 (3)	C13—C18	1.527 (3)
C3—C4	1.521 (4)	C13—C17	1.532 (3)
C3—H3B	0.9800	C13—C14	1.539 (3)
C4—C5	1.537 (3)	C14—C15	1.532 (3)
C4—H4B	0.9700	C14—H14	0.9800
C4—H4C	0.9700	C15—C16	1.515 (3)
C5—C6	1.525 (3)	C15—H15A	0.9700
C5—C10	1.551 (2)	C15—H15B	0.9700
C5—H5A	0.9800	C16—C17	1.512 (4)
C6—C7	1.523 (3)	C17—H17A	0.9700
C6—H6A	0.9700	C17—H17B	0.9700
C6—H6B	0.9700	C18—H18A	0.9600
C7—C8	1.528 (2)	C18—H18B	0.9600
C7—H7A	0.9700	C18—H18C	0.9600
C7—H7B	0.9700	C19—H19A	0.9700
C8—C14	1.524 (2)	C19—H19B	0.9700
C8—C9	1.542 (3)		
			
C19—O1—C2	109.44 (13)	C1—C10—C9	113.66 (15)
C2—O2—H2A	109.5	C1—C10—C19	100.18 (14)
C3—O3—H3A	109.5	C9—C10—C19	115.84 (14)
C2—C1—C10	101.51 (15)	C1—C10—C5	107.06 (15)
C2—C1—H1A	111.5	C9—C10—C5	109.94 (13)
C10—C1—H1A	111.5	C19—C10—C5	109.50 (15)
C2—C1—H1B	111.5	C12—C11—C9	113.59 (15)
C10—C1—H1B	111.5	C12—C11—H11A	108.8
H1A—C1—H1B	109.3	C9—C11—H11A	108.8
O2—C2—O1	108.94 (16)	C12—C11—H11B	108.8
O2—C2—C1	111.31 (17)	C9—C11—H11B	108.8
O1—C2—C1	103.11 (15)	H11A—C11—H11B	107.7
O2—C2—C3	114.50 (17)	C13—C12—C11	110.12 (16)
O1—C2—C3	107.03 (17)	C13—C12—H12A	109.6
C1—C2—C3	111.22 (16)	C11—C12—H12A	109.6
O3—C3—C4	111.86 (18)	C13—C12—H12B	109.6
O3—C3—C2	106.94 (18)	C11—C12—H12B	109.6
C4—C3—C2	109.48 (16)	H12A—C12—H12B	108.2
O3—C3—H3B	109.5	C12—C13—C18	110.98 (17)
C4—C3—H3B	109.5	C12—C13—C17	115.81 (17)
C2—C3—H3B	109.5	C18—C13—C17	107.28 (17)
C3—C4—C5	114.94 (17)	C12—C13—C14	108.43 (16)
C3—C4—H4B	108.5	C18—C13—C14	113.29 (16)
C5—C4—H4B	108.5	C17—C13—C14	100.79 (15)
C3—C4—H4C	108.5	C8—C14—C15	119.80 (17)
C5—C4—H4C	108.5	C8—C14—C13	113.53 (13)
H4B—C4—H4C	107.5	C15—C14—C13	103.60 (15)
C6—C5—C4	109.85 (17)	C8—C14—H14	106.3
C6—C5—C10	112.35 (16)	C15—C14—H14	106.3
C4—C5—C10	110.84 (14)	C13—C14—H14	106.3
C6—C5—H5A	107.9	C16—C15—C14	103.53 (19)
C4—C5—H5A	107.9	C16—C15—H15A	111.1
C10—C5—H5A	107.9	C14—C15—H15A	111.1
C7—C6—C5	112.59 (18)	C16—C15—H15B	111.1
C7—C6—H6A	109.1	C14—C15—H15B	111.1
C5—C6—H6A	109.1	H15A—C15—H15B	109.0
C7—C6—H6B	109.1	O4—C16—C17	125.6 (2)
C5—C6—H6B	109.1	O4—C16—C15	125.4 (3)
H6A—C6—H6B	107.8	C17—C16—C15	108.98 (18)
C6—C7—C8	111.69 (15)	C16—C17—C13	102.17 (17)
C6—C7—H7A	109.3	C16—C17—H17A	111.3
C8—C7—H7A	109.3	C13—C17—H17A	111.3
C6—C7—H7B	109.3	C16—C17—H17B	111.3
C8—C7—H7B	109.3	C13—C17—H17B	111.3
H7A—C7—H7B	107.9	H17A—C17—H17B	109.2
C14—C8—C7	111.37 (14)	C13—C18—H18A	109.5
C14—C8—C9	108.93 (15)	C13—C18—H18B	109.5
C7—C8—C9	109.80 (16)	H18A—C18—H18B	109.5
C14—C8—H8A	108.9	C13—C18—H18C	109.5
C7—C8—H8A	108.9	H18A—C18—H18C	109.5
C9—C8—H8A	108.9	H18B—C18—H18C	109.5
C8—C9—C11	112.89 (15)	O1—C19—C10	105.95 (16)
C8—C9—C10	111.57 (15)	O1—C19—H19A	110.5
C11—C9—C10	113.97 (14)	C10—C19—H19A	110.5
C8—C9—H9	105.9	O1—C19—H19B	110.5
C11—C9—H9	105.9	C10—C19—H19B	110.5
C10—C9—H9	105.9	H19A—C19—H19B	108.7
			
C19—O1—C2—O2	−142.16 (15)	C4—C5—C10—C1	−60.1 (2)
C19—O1—C2—C1	−23.84 (19)	C6—C5—C10—C9	52.7 (2)
C19—O1—C2—C3	93.54 (17)	C4—C5—C10—C9	176.02 (17)
C10—C1—C2—O2	158.11 (16)	C6—C5—C10—C19	−75.65 (19)
C10—C1—C2—O1	41.46 (17)	C4—C5—C10—C19	47.7 (2)
C10—C1—C2—C3	−72.9 (2)	C8—C9—C11—C12	50.3 (2)
O2—C2—C3—O3	64.1 (2)	C10—C9—C11—C12	178.96 (17)
O1—C2—C3—O3	−175.03 (16)	C9—C11—C12—C13	−54.3 (2)
C1—C2—C3—O3	−63.1 (2)	C11—C12—C13—C18	−67.1 (2)
O2—C2—C3—C4	−174.50 (18)	C11—C12—C13—C17	170.30 (18)
O1—C2—C3—C4	−53.7 (2)	C11—C12—C13—C14	57.9 (2)
C1—C2—C3—C4	58.3 (2)	C7—C8—C14—C15	−59.0 (2)
O3—C3—C4—C5	75.6 (2)	C9—C8—C14—C15	179.72 (16)
C2—C3—C4—C5	−42.7 (2)	C7—C8—C14—C13	177.94 (17)
C3—C4—C5—C6	169.86 (18)	C9—C8—C14—C13	56.7 (2)
C3—C4—C5—C10	45.1 (2)	C12—C13—C14—C8	−61.8 (2)
C4—C5—C6—C7	−176.24 (17)	C18—C13—C14—C8	61.8 (2)
C10—C5—C6—C7	−52.4 (2)	C17—C13—C14—C8	176.12 (17)
C5—C6—C7—C8	54.4 (3)	C12—C13—C14—C15	166.64 (15)
C6—C7—C8—C14	−177.36 (19)	C18—C13—C14—C15	−69.71 (19)
C6—C7—C8—C9	−56.6 (2)	C17—C13—C14—C15	44.6 (2)
C14—C8—C9—C11	−49.58 (18)	C8—C14—C15—C16	−156.82 (17)
C7—C8—C9—C11	−171.78 (16)	C13—C14—C15—C16	−29.09 (19)
C14—C8—C9—C10	−179.45 (14)	C14—C15—C16—O4	−179.7 (2)
C7—C8—C9—C10	58.35 (18)	C14—C15—C16—C17	2.5 (2)
C2—C1—C10—C9	−166.58 (15)	O4—C16—C17—C13	−152.8 (2)
C2—C1—C10—C19	−42.37 (17)	C15—C16—C17—C13	25.0 (2)
C2—C1—C10—C5	71.82 (17)	C12—C13—C17—C16	−158.74 (18)
C8—C9—C10—C1	−176.07 (14)	C18—C13—C17—C16	76.7 (2)
C11—C9—C10—C1	54.6 (2)	C14—C13—C17—C16	−42.0 (2)
C8—C9—C10—C19	68.68 (19)	C2—O1—C19—C10	−3.4 (2)
C11—C9—C10—C19	−60.6 (2)	C1—C10—C19—O1	28.71 (17)
C8—C9—C10—C5	−56.09 (19)	C9—C10—C19—O1	151.40 (15)
C11—C9—C10—C5	174.61 (17)	C5—C10—C19—O1	−83.62 (18)
C6—C5—C10—C1	176.60 (15)		

### Hydrogen-bond geometry (Å, °)

**Table:**

*D*—H···*A*	*D*—H	H···*A*	*D*···*A*	*D*—H···*A*
O2—H2A···O1^i^	0.82	2.03	2.8088 (19)	158

Symmetry codes: (i) −*x*, *y*+1/2, −*z*+1.
